# Unequal error protection technique for video streaming over MIMO-OFDM systems

**DOI:** 10.1371/journal.pone.0210112

**Published:** 2019-01-15

**Authors:** Jaeyoung Park, Yusik Yang, Jaekwon Kim

**Affiliations:** Division of Computer and Telecommunications Engineering, Yonsei University, Wonju, Gangwon-do, Republic of Korea; King Abdulaziz University, SAUDI ARABIA

## Abstract

In this paper, a novel unequal error protection (UEP) technique is proposed for video streaming over multiple-input multiple-output orthogonal frequency-division multiplexing (MIMO-OFDM) systems. Based on the concept of hierarchical quadrature amplitude modulation (HQAM) UEP and multi-antenna UEP, the proposed technique combines the relative protection levels (PLs) of constellation symbols and the differentiated PLs of the transmit antennas. In the proposed technique, standard square quadrature amplitude modulation (QAM) constellations are used instead of HQAM so that the QAM mapper at the transmitter side and the soft decision calculation at the receiver side remain unchanged, but the UEP benefit of HQAM is retained. The superior performance of the proposed technique is explained by the improved connections between data with various priorities and data paths with various PLs. The assumed video compression method is H.264/AVC, which is known to be commercially successful. The IEEE802.16m system is adopted as a data transmission system. With the aid of realistic simulations in strict accordance with the standards of IEEE802.16m systems and H.264/AVC video compression systems, the proposed technique *HQAM-multi-antenna UEP* is shown to improve the video quality significantly for a given average bit error rate when compared with previous techniques.

## 1 Introduction

Video streaming services, such as video conferencing, multimedia messaging, and video telephony, operating under unstable wireless channel environments present significant challenges [1]. To overcome unstable channel environments, various network layer unequal error protection (UEP) techniques have been proposed [2], [3], [4]. However, these network layer techniques do not directly consider the unstable channels of the physical (PHY) layer but use various abstraction methods of the wireless channels. Another classical UEP implementation method is the use of channel coding with different redundancies. An efficient implementation of numerous different redundancies involves performing puncturing after channel coding with a high redundancy as in [5] and [6], which used convolutional code and turbo code, respectively. The work in [5] considered only flat fading channels; these are not suitable for broadband communications, which are considered in this paper. Broadband wireless channels were considered in video streaming in [6] and [7]; the latter work included the details of Mobile WiMAX systems. In [8], a multi-user scenario is considered, and multi-user diversity was exploited to improve video quality. In the previous works [6]–[8], however, only single-antenna systems were considered.

Multiple antennas at both transmitter and receiver sides can increase either the data transmission speed or the transmission reliability without requiring additional transmission power and (or) spectral bandwidth [9]. Owing to these advantages, various multiple-antenna techniques have been included in the IEEE802.16m systems [10] and LTE-advanced (LTE-A) systems [11]. UEP techniques that use multiple antennas are important for video streaming over these currently popular cellular systems. Jubran *et* *al*. proposed a multi-antenna UEP technique, assuming orthogonal space-time block coding (OSTBC), which is known to increase the transmission reliability but offers rather low data transmission speed [12]. It is known that the data transmission speed of OSTBCs is no greater than that of single-antenna systems. Song *et* *al*. considered a spatially multiplexed (SM) multiple-antenna technique that enables high-speed data transmission suitable for video transmission [13]. A video stream with higher priority was assigned to a transmitter antenna with higher reliability. Song *et* *al*. adopted linear detection methods, which offer the reliability information of transmitter antennas as post-detection signal-to-noise ratio (SNR); however, the adopted linear detection techniques are known to offer significantly degraded error performance when compared with joint detection methods. Abot *et* *al*. also considered SM multiple-input multiple-output (MIMO) systems, decomposing a multi-antenna channel into numerous virtual single-antenna channels [14]. However, the channel state information (CSI) should be fed back from the receiver to the transmitter for the channel decomposition in [14]. An SM multiple-antenna system with joint signal detection at the receiver side is a multiple-antenna technique that enables both high-speed data transmission and good error performance without the burdensome feedback of CSI.

Recently, Yun *et* *al*. proposed a UEP video-streaming technique that uses SM multiple antennas and a joint signal detection method [15]. The challenge of UEP implementation using joint detection is how to decide the reliabilities of transmitter antennas when all the signals are detected jointly. It was shown that multi-antenna UEP can be implemented by exploiting the structure of a joint detection method *QR-LRL* [16], and it was demonstrated that the joint-detection-based multi-antenna UEP dramatically outperforms the linear-detection-based UEP in terms of video quality. Yun *et* *al*. also showed that the antenna reliability assessment technique for the QR-LRL is also valid for the optimal maximum likelihood (ML) detection [17]. The improved video quality by using ML, however, is achieved at the expense of increased complexity at the receiver owing to the exhaustive search in ML detection. The prohibitive complexity of ML can be mitigated to some degree [18], but its complexity is still too high for practical use.

Hierarchical quadrature amplitude modulation (HQAM) is a well-known technique used to implement UEP. Barmada *et* *al*. showed that HQAM can be successfully combined with UEP based on channel coding with different redundancies [19]. More robust constellation points were assigned to higher-priority video data whose transmission speed is reduced owing to the high redundancy. Chang *et* *al*. applied the HQAM technique to wireless video transmission over additive white Gaussian noise (AWGN) channel [20]. Li *et* *al*. used the idea of HQAM in conjunction with orthogonal frequency-division multiplexing (OFDM) to provide video-streaming services over frequency-selective channels [21]. To improve the reconstructed video quality, Li *et* *al*. utilized both the SNRs of the OFDM sub-channels and the HQAM. However, the technique proposed by Li *et al*., which is intended for single-antenna systems, cannot be applied directly to multiple-antenna systems, because the evaluation method for the sub-channel SNRs cannot be used for multiple-antenna systems. Furthermore, normal square quadrature amplitude modulations (QAMs) have been adopted in the contemporary cellular mobile systems such as IEEE802.16m and LTE-A systems; thus, the previous HQAM UEP techniques cannot be applied in their present forms.

In this paper, a novel HQAM-multi-antenna UEP technique is proposed, considering realistic multiple-antenna channels. Pilot insertion, channel coding for error correction, resource allocation, and precoding at the transmitter side are performed in strict accordance with the IEEE802.16m system standard [10]. Channel estimation at the pilot tone positions is performed using the least square (LS) method; subsequently, the channel gains at data positions are obtained via the linear interpolation technique, which is used widely in contemporary systems. The proposed technique combines the different error protection properties of QAM constellation symbols and the antenna reliability information, using both multi-antenna UEP [15], [17] and the concept of HQAM UEP [20], [21]. The standard square QAM instead of HQAM is adopted in the proposed technique; thus, the constellation in the standard need not be modified, but the benefit of HQAM is retained. We note that the spatial streams are different from the transmit antennas when precoding is used as in IEEE802.16m systems. However, we use the two terms interchangeably because precoding is not an issue in this study and the term “transmit antenna” is more easily visualized. The simulation will demonstrate the successful combination of the HQAM concept with the previous multi-antenna UEP, which offers improved video quality. A short abstract form of this paper was published as a conference paper [22]; in the present extended paper, a more detailed explanation of the proposed technique and more extensive experiment results are provided. This extended version also includes the PHY layer wireless resource usage of the previous and proposed UEP techniques in order to explain the performance improvement achieved using the proposed UEP technique.

The remainder of the paper is organized as follows. Section 2 describes a cross-layer system including the H.264/AVC application layer [1] and the IEEE802.16m physical layer [10]. Section 3 summarizes the previous two main UEP techniques: HQAM UEP and multi-antenna UEP. A novel UEP video-streaming technique exploiting both multi-antenna UEP and the idea of HQAM is described in Section 4. The improvement in video peak signal-to-noise ratio (PSNR) performance achieved using the proposed technique is confirmed in Section 5 with the aid of an extensive set of computer simulations. Section 6 presents the conclusions.

## 2 APP-PHY cross-layer system model

**Fig 1:**
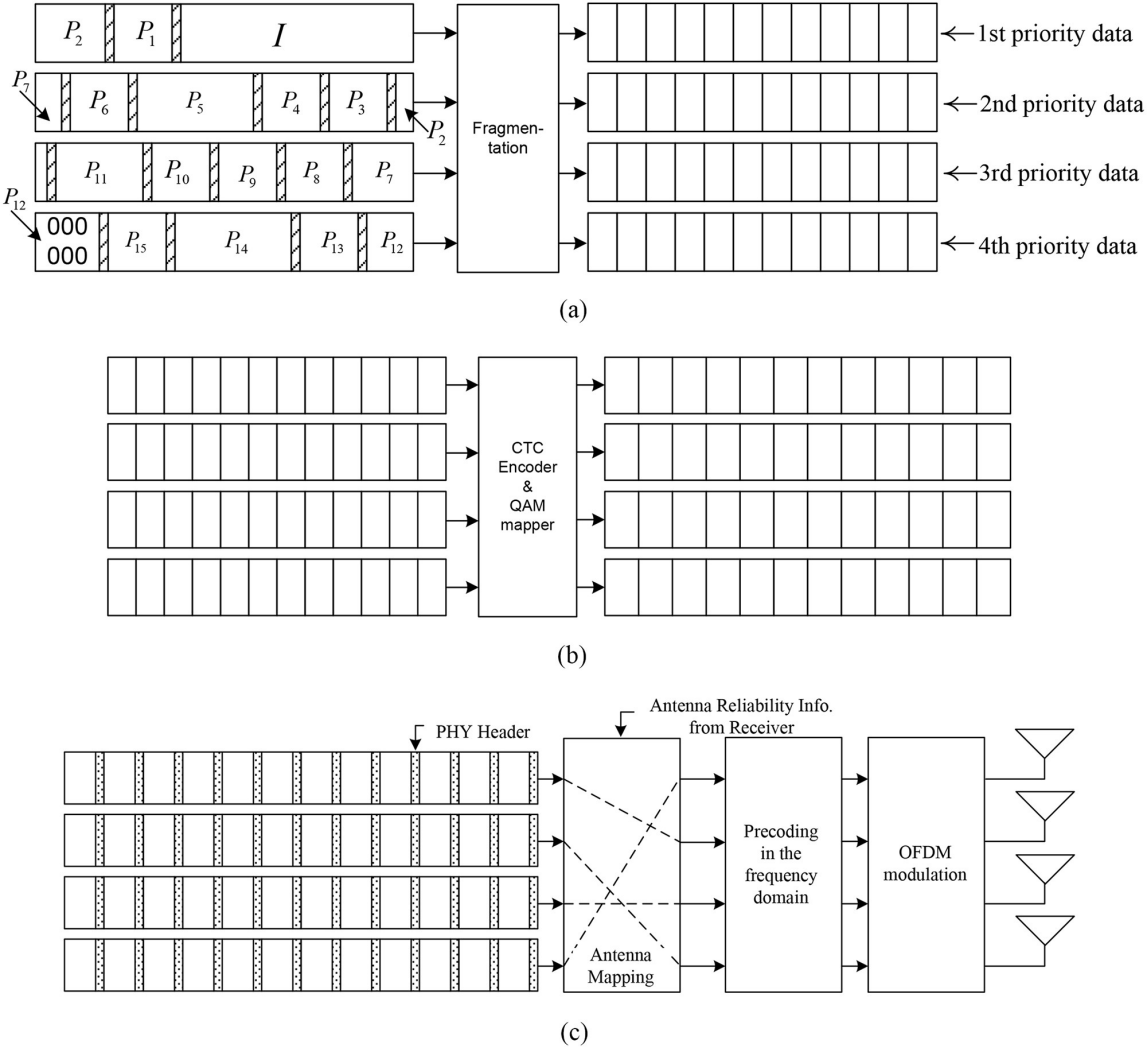
APP-PHY cross-layer system model (a) H.264/AVC video encoding, CRC encoding, prioritization, fragmentation; (b) convolutional turbo encoding, constellation mapping; (c) antenna mapping, precoding, OFDM modulation.

This section describes the application-physical (APP-PHY) cross-layer system illustrated in Fig 1. The APP layer is H.264/AVC, which produces network abstraction layer (NAL) unit streams [1]. In this study, H.264/AVC encodes a group of pictures (GOP) into 16 NAL units (an NAL unit corresponds to a picture), and cyclic redundancy check (CRC) parity bits are added to each NAL unit. The white area in the left side of Fig 1(a) indicates an NAL unit, and the shaded area at the end of each NAL unit indicates the CRC bits. The 16 NAL units are thereafter prioritized and zero-padded so that a GOP (after the CRC encoding) is an integer multiple of 4 (the number of priority levels) times the PHY packet size; here, the PHY packet size represents the size before PHY layer channel encoding with the convolutional turbo code (CTC). If the number of priority levels is changed, the prioritization in Fig 1(a) should be changed accordingly. Assuming that a GOP is composed of one I-picture and 15 P-pictures as shown in Fig 1(a), the prioritization is straightforward. Thus, the first quarter of the GOP that includes the I-picture is of the highest priority, the second quarter is of the second highest priority, and so on. Each prioritized NAL stream is divided to be of PHY packet size and stored in the data buffer of the corresponding priority level, as shown on the right side of Fig 1(a). Each PHY payload data packet is channel-encoded using CTC, and is mapped to the QAM symbols as shown in Fig 1(b). The four PHY packet streams are assigned to appropriate spatial streams using the reliability information from the receiver. Precoding in the frequency domain and OFDM modulation are performed. Finally, multiple packets of different priorities are transmitted simultaneously from multiple antennas.

Assuming four transmitter and four receiver antennas, the relationship between the transmitted signals and the received signals in the PHY layer is described in the frequency domain as follows:

$$\begin{array}{c} y_{k}=H_{k}x_{k}+z_{k} \end{array}$$ (1)

where **x**_*k*_ = [*x*_1,*k*_ *x*_2,*k*_ *x*_3,*k*_ *x*_4,*k*_]^*T*^, *x*_*i*,*k*_ is the transmitted signal from the *i*th transmitter antenna, **y**_*k*_ = [*y*_1,*k*_ *y*_2,*k*_ *y*_3,*k*_ *y*_4,*k*_]^*T*^, *y*_*j*,*k*_ denotes the received signal at the *j*th receiver antenna, **H**_*k*_ is a 4 × 4 matrix in which *h*_*ji*,*k*_ is the channel gain between the *i*th transmitter antenna and the *j*th receiver antenna, **z**_*k*_ = [*z*_1,*k*_ *z*_2,*k*_ *z*_3,*k*_ *z*_4,*k*_]^*T*^, *z*_*j*,*k*_ denotes the additive noise at the *j*th receiver antenna, and the subscript *k* denotes the OFDM subcarrier index allocated to the target user by a scheduling algorithm. In this study, we do not consider scheduling for multiple users; instead, we allocate sufficient wireless resource to a single target user considering the resource allocation unit of WirelessMAN-Advanced system standard.

At the receiver side, channel gains are estimated using the linear interpolation method; thus, estimation errors are included in the considered systems, and the PHY packets of different priorities are detected using a joint detection technique such as QR-LRL, which uses the estimated channel gains. The stream reliability is calculated, and subsequently, the reliability order information is passed to the transmitter side to be used in the antenna mapping in Fig 1(c). Stream reliability assessment technique and the assumed joint signal detection technique proposed by Yun *et* *al*. are adopted, and are briefly reviewed in Section 3.1. The soft decisions calculated using the joint detection technique are passed to the CTC decoder for error correction. The CRC is thereafter checked for each NAL unit, and the acknowledgement (ACK) or negative ACK (NACK) result is passed to the APP layer to be used possibly for either retransmission request or error concealment. Frame-copy error concealment is used and retransmission is not allowed in the systems considered in the study.

Notably, we deal with single-user MIMO systems, indicating that a resource block (allocated to a specific user) occupies all the spatial streams, and that the APP layer video data (prioritized and divided) originates from a single user.

## 3 Previous UEP techniques

### 3.1 UEP using multiple antennas

Yun *et* *al*. proposed a UEP technique for multiple-antenna systems using the joint signal detection technique QR-LRL, which is known to achieve a near-ML performance [15]. Using QR decomposition **H**_*k*_ = [**h**_1,*k*_ **h**_2,*k*_ **h**_3,*k*_ **h**_4,*k*_] = **Q**_*k*_ **R**_*k*_ where **Q**_*k*_ is a unitary matrix and **R**_*k*_ is an upper triangular matrix, Eq (1) is modified as follows:

$$\begin{array}{c} {\tilde{y}}_{k}=R_{k}x_{k}+{\tilde{z}}_{k} \end{array}$$ (2)

where ${\tilde{y}}_{k}=Q_{k}^{H}y_{k}$, ${\tilde{z}}_{k}=Q_{k}^{H}z_{k}$, and (.)^*H*^ denotes the Hermitian transpose. The QR-LRL signal detection generates a set of candidate vectors *S*_QR−LRL_ as presented in Table 1, where ${\tilde{y}}_{j,k}$ is the *j*th entry of ${\tilde{y}}_{k}$, Ω denotes the set of constellation points, |Ω| denotes its cardinality, and *D*[.] denotes the slicer for the adopted constellation. Although there is generally no order of elements in a set, Ω(*i*) denotes the *i*th element of the constellation set Ω in this study. In QR-LRL detection, the columns of **H**_*k*_ are reordered as follows:

$$\begin{array}{c} H_{k,\text{ord}}=\left[h_{(1),k}\;h_{(2),k}\;h_{(3),k}\;h_{(4),k}\right] \end{array}$$ (3)

where **h**_(*i*),*k*_, *i* = 1, 2, 3, 4 denotes the *i*th column vector after the ordering. The ordering in Eq (3) is conducted so that the diagonal entries of the matrix **R**_*k*,ord_, which are obtained through the QR decomposition of **H**_*k*,ord_, satisfy the following conditions:

$$\begin{array}{c} r_{(44),k}<r_{(11),k}<r_{(22),k}<r_{(33),k} \end{array}$$ (4)

where *r*_(*ji*),*k*_ denotes *ji*th entry of **R**_*k*,ord_.

**Table 1:** Generation of a candidate vector set.

*S*_QR − LRL_ ← {.}; initialization
For *c*=1:|Ω|
*x*_(4),*k*_ ← Ω(*c*)
$x_{(3),k}\leftarrow D\left[\left({\tilde{y}}_{3,k}-\sum_{l=4}^{4}r_{(3l),k}x_{(l),k}\right)/r_{(33),k}\right]$
$x_{(2),k}\leftarrow D\left[\left({\tilde{y}}_{2,k}-\sum_{l=3}^{4}r_{(2l),k}x_{(l),k}\right)/r_{(22),k}\right]$
$x_{(1),k}\leftarrow D\left[\left({\tilde{y}}_{1,k}-\sum_{l=2}^{4}r_{(1l),k}x_{(l),k}\right)/r_{(11),k}\right]$
*S*_QR−LRL_ ← *S*_QR−LRL_ ∪ {[*x*_(1),*k*_ *x*_(2),*k*_ *x*_(3),*k*_ *x*_(4),*k*_]^*T*^}
End

Yun *et* *al*. showed that the reliability order of multiple transmitter antennas is statistically the same as Eq (4) when QR-LRL detection is used, i.e., the transmitter antenna that corresponds to *r*_(33),*k*_ (or **h**_(3),*k*_) shows the highest reliability, the antenna that corresponds to *r*_(11),*k*_ (or **h**_(1),*k*_) shows the third highest reliability, and so on [15]. Note that the antenna reliability information should be calculated for each OFDM subcarrier index *k*. The same reliability ordering was shown to be valid even when ML signal detection is used [17]. The candidate vectors in the set *S*_QR−LRL_ are used for the calculation of soft decisions fed to the CTC decoder of WirelessMAN-Advanced systems.

### 3.2 UEP using hierarchical QAM

**Fig 2:**
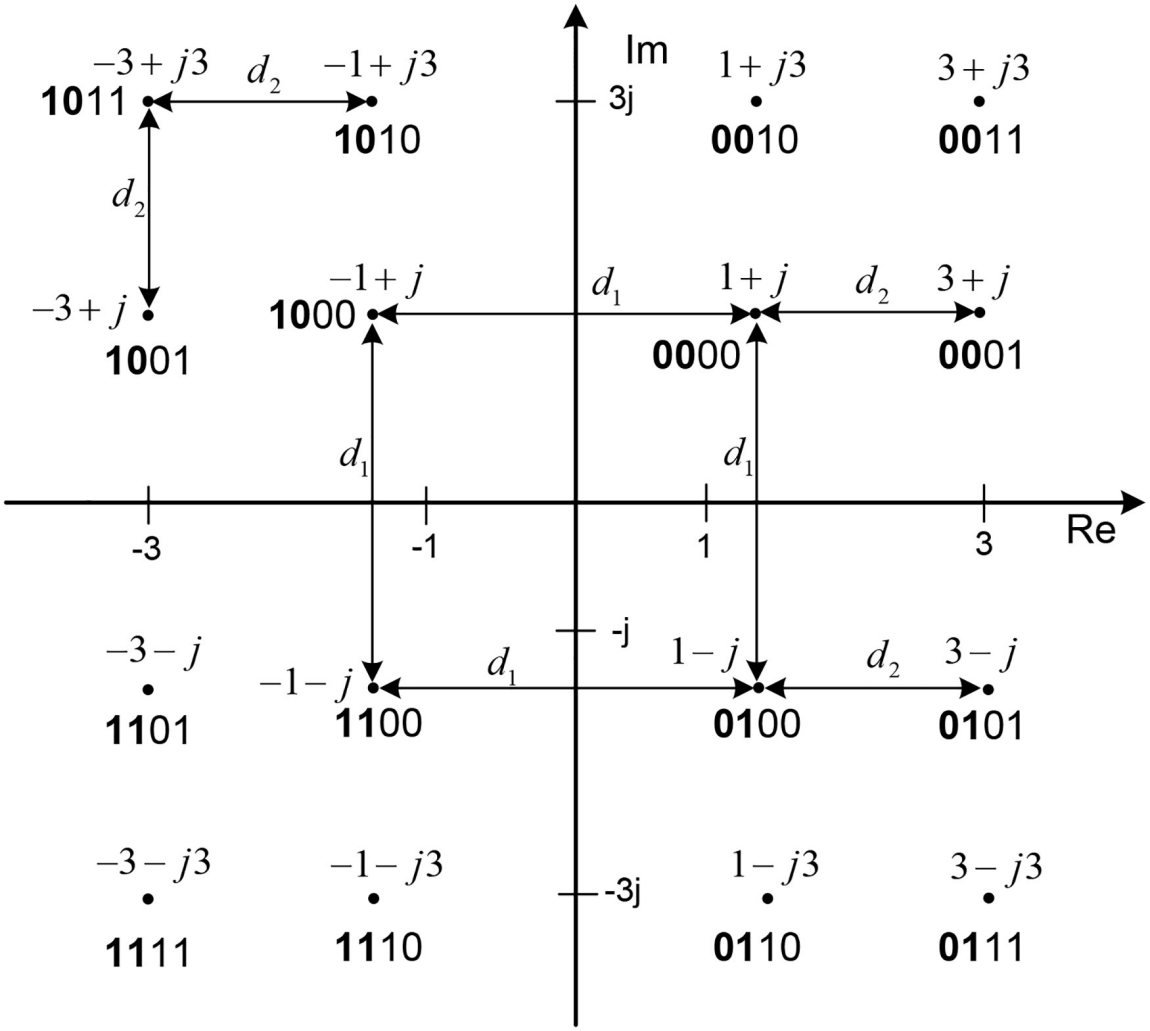
Hierarchical 16-QAM constellation. The two MSB bits are bold-faced.

The concept of HQAM constellation can also be used to implement UEP. A hierarchical 16-QAM constellation is illustrated in Fig 2, where the two most significant bits (MSBs) are bold-faced. From Fig 2, it can be observed that the minimum distance between symbols with two different MSBs is *d*_1_, and that for two different least significant bits (LSBs) is *d*_2_. With the parameter *α* = *d*_1_/*d*_2_, the bit error probability of the MSBs and LSBs is expressed as follows:

$$\begin{array}{c} P_{e}\left(\text{MSBs}\right)=\frac{1}{2}Q(\sqrt{\frac{2\gamma {\left(\alpha +2\right)}^{2}}{{\left(\alpha +1\right)}^{2}+1}})+\frac{1}{2}Q(\sqrt{\frac{2\gamma \alpha^{2}}{{\left(\alpha +1\right)}^{2}+1}}) \end{array}$$ (5)

$$\begin{array}{ccc} P_{e}\left(\text{LSBs}\right) & = & \frac{1}{2}Q(\sqrt{\frac{2\gamma }{{\left(\alpha +1\right)}^{2}+1}})+\frac{1}{2}Q(\sqrt{\frac{4\gamma (2\alpha^{2}+5\alpha +4)}{{\left(\alpha +1\right)}^{2}+1}})\\ & & -\frac{1}{2}Q(\sqrt{\frac{4\gamma (2\alpha^{2}-5\alpha +4)}{{\left(\alpha +1\right)}^{2}}}) \end{array}$$ (6)

where *γ* = *E*_*b*_/*N*_0_. As the parameter *α* increases, the protection of two MSBs become stronger, but simultaneously the LSBs become more erroneous owing to the reduced *d*_2_. Chang *et* *al*. applied the HQAM technique to wireless video transmission, considering AWGN channels [20]. However, when the spectral bandwidth is as large as 20 MHz or 40 MHz as in contemporary communication systems [10], [11], realistic wireless channels are frequency-selective fading channels. Li *et* *al*. used the idea of HQAM in conjunction with OFDM to enable wireless video transmission over frequency-selective channels [21]. The bit level SNR at the *k*th subcarrier of OFDM systems is evaluated as follows:

$$\begin{array}{c} \gamma_{k}=\frac{{|H_{k}|}^{2}E_{b}}{N_{0}} \end{array}$$ (7)

where *H*_*k*_ is the gain of the *k*th subchannel of OFDM systems, and *E*_*b*_ and *N*_0_ denote the bit energy and noise energy, respectively. Replacing *γ* in Eqs (5) and (6) with *γ*_*k*_ shown above, Li *et al*. successfully combined the ideas of HQAM and OFDM. The subcarriers were classified into two categories, i.e., high quality (HQ) and low quality (LQ), based on the SNR evaluation method Eq (7). Video streams with high significance are transmitted over HQ subcarriers, and similarly, video streams with low significance are transmitted over LQ subcarriers, where each HQ or LQ subcarrier uses the normal square QAM constellation. If the number of HQ subcarriers is not sufficient to accommodate the video streams with high significance, the LQ subcarriers are again divided into two categories: medium quality (MQ) and LQ. Subsequently, the HQAM UEP is applied to MQ subcarriers so that the MSBs of the MQ subcarriers are used for a part of a video stream with high significance.

The quality assessment of subcarriers in Eq (7) holds for single-antenna systems, because simple one-tap equalization is optimal for OFDM systems with a single antenna. When multiple antennas are used as in IEEE802.16m systems [10] and LTE-A systems [11], unfortunately, the channel quality assessment method in Eq (7) cannot be adopted. Consequently, we require a novel technique that considers the HQAM UEP, multiple-antenna systems, and the structure of the adopted joint detection method for successful video streaming over contemporary multi-antenna communication systems. We also note that all the previous HQAM UEP techniques in their present forms cannot be applied to contemporary mobile systems such as IEEE802.16m and LTE-A systems because normal square QAMs have been adopted in the standards.

## 4 Proposed HQAM-multi-antenna UEP technique

In this section, the error performance of QAM is briefly reviewed first. Subsequently, a novel UEP technique that utilizes the concepts of HQAM UEP and multi-antenna UEP is proposed.

### 4.1 Review of QAM performance

When the HQAM parameter *α* = 1, the two distances in Fig 2 become equal, i.e., *d*_1_ = *d*_2_ = *d* = 2, resulting in the normal rectangular QAM constellation. The QAM symbols corresponding to *LSB* = 0 (i.e., the fourth bit is 0) are −1 + *j*3, −1 + *j*, −1 − *j*, −1 − *j*3, 1 + *j*3, 1 + *j*, 1 − *j*, and 1 − *j*3. When −1 + *j*3 is considered, the closest symbol with flipped LSB, i.e., *LSB* = 1, is −3 + *j*3, and the distance between the two symbols is *d*. The minimum distance for all the other seven symbols is readily shown to be also *d*. Similarly, it can be shown that the minimum distance between symbols with flipped third bits is also *d*. In summary, the two LSBs (the third and fourth bits) are equally protected, which is characterized by the minimum distance of *d*.

The QAM symbols corresponding to *MSB* = 0, i.e., the first bit is 0, are 1 + *j*3, 1 + *j*, 1 − *j*, 1 − *j*3, 3 + *j*3, 3 + *j*, 3 − *j*, and 3 − *j*3. The minimum distance between the symbols with flipped MSB is *d* when the four symbols 1 + *j*3, 1 + *j*, 1 − *j*, and 1 − *j*3 are considered, but the minimum distance is 2*d* when the four symbols 3 + *j*3, 3 + *j*, 3 − *j*, and 3 − *j*3 are considered. It can also be shown that the minimum distance between symbols with flipped second bits is *d* for four symbols and 2*d* for the other four symbols. In summary, even when *d*_1_ = *d*_2_ = *d*, the first two bits (MSBs) are sometimes protected more than the last two bits (LSBs). Thus, the performance difference between MSBs and LSBs is crucial when realistic systems are considered, which include rather strong error correction techniques such as CTC, channel estimation error owing to fixed pattern and linear interpolation process, realistic resource allocation, and realistic wireless channel realizations.

As will be shown in the simulation section, the average performance difference between the MSBs and LSBs even with *α* = 1 is significant when realistic channel coding, pilot pattern insertion, precoding, and resource allocation scheme are used in accordance with the WirelessMAN-Advanced standard. Using a normal QAM constellation is beneficial because the parameter *α* need not be calculated to satisfy the desired performance requirement. Furthermore, the QAM mapper at the transmitter and the soft decision calculation at the receiver need not be changed. Combining the multi-antenna UEP in Section 3.1 and the concept of HQAM UEP in Section 3.2, we propose the resource mapping method in Table 2. As shown in Table 2, the highest protection is achieved by the PHY packets that correspond to the 2 MSBs of QAM symbols transmitted from the first reliable antenna. It can be observed that the two MSBs of the fourth reliable antenna are assumed to provide stronger protection than the two LSBs of the first reliable antenna in the proposed technique. We reiterate that the HQAM parameter *α* is assumed to be 1 in the proposed technique.

**Table 2:** Resource mapping for different protection levels (PLs) in the proposed HQAM-multi-antenna UEP technique.

Protection Level (PL)	Used PHY Layer Resource
1	2 MSBs of the 1st reliable antenna symbols
2	2 MSBs of the 2nd reliable antenna symbols
3	2 MSBs of the 3rd reliable antenna symbols
4	2 MSBs of the 4th reliable antenna symbols
5	2 LSBs of the 1st reliable antenna symbols
6	2 LSBs of the 2nd reliable antenna symbols
7	2 LSBs of the 3rd reliable antenna symbols
8	2 LSBs of the 4th reliable antenna symbols

### 4.2 Proposed UEP technique

**Fig 3:**
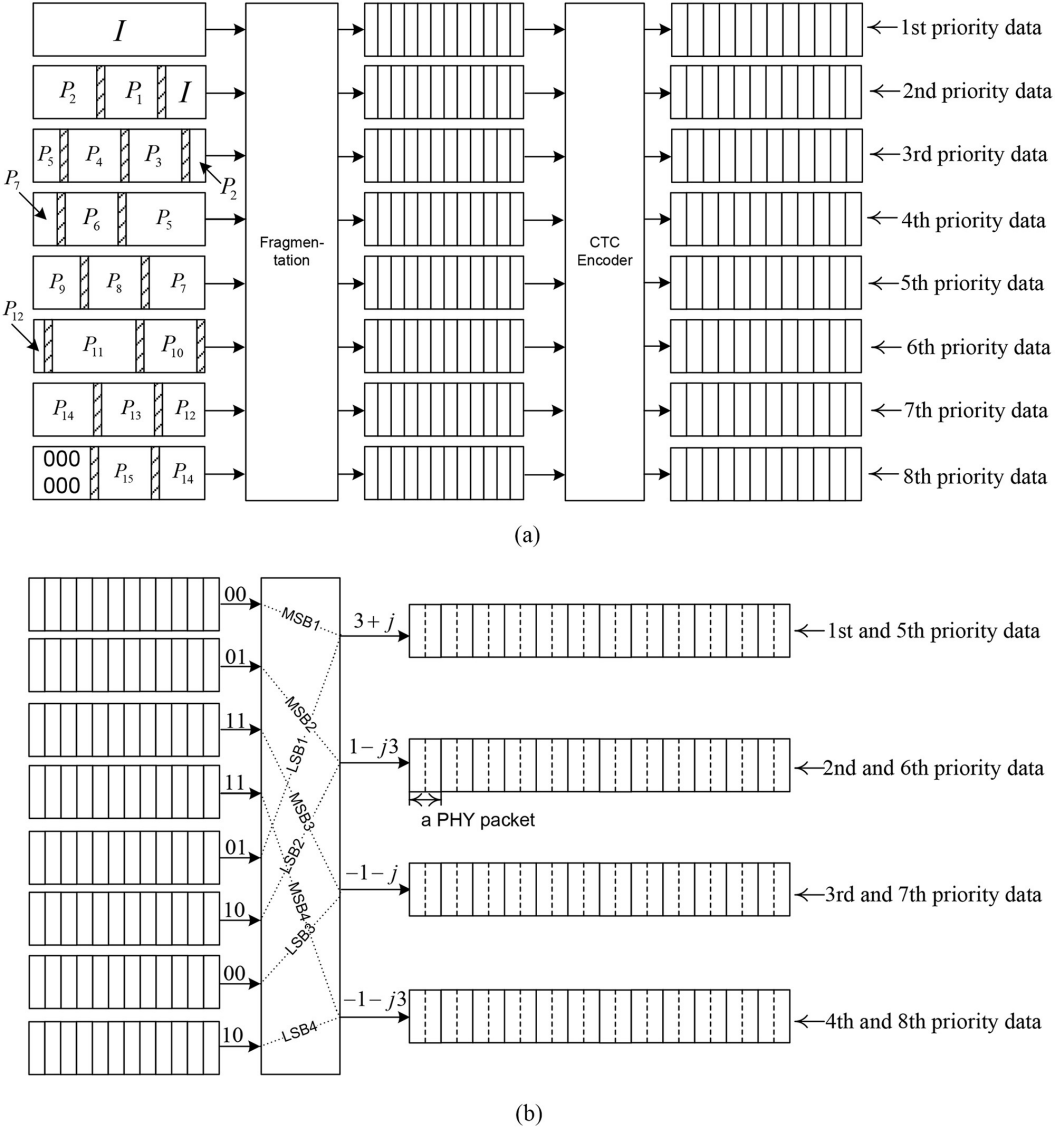
Proposed HQAM-multi-antenna UEP technique (a) H.264/AVC video encoding, CRC encoding, prioritization, fragmentation, convolutional turbo encoding; (b) conventional rectangular QAM mapping, PHY packetization.

Fig 3 illustrates the proposed HQAM-multi-antenna UEP technique when applied to WirelessMAN-Advanced systems. As shown in Fig 3(a), H.264/AVC video is segmented into 8 priority levels instead of 4 levels as in the multi-antenna UEP in Fig 1(a); each video segment of a particular priority is fragmented into blocks that are half the size of a PHY packet, and thereafter, each block is CTC encoded. Thus, the top block stream on the right side of Fig 3(a) is of the highest priority, the second block stream from the top is of the second highest priority, and so on. Fig 3(b) shows an example of QAM mapping, in which two block streams are mapped onto the MSBs and LSBs of QAM symbols. *MSBi* and *LSBi*, *i* = 1, 2, 3, 4 are mapped onto symbols transmitted from the transmitter antenna of the *i*th reliability. For example, when MSB2 and LSB2 are 01 and 10, respectively, as in Fig 3(b), the four bits are mapped onto a complex symbol 1 − *j*3, according to the QAM constellation in Fig 2, and the complex symbol is transmitted from the second reliable antenna. The PHY packet streams after the QAM mapping are transmitted from the transmitter antennas as in the previous multi-antenna UEP in Fig 1(c). Owing to the QAM mapping method in the proposed technique, the *i*th most reliable antenna transmits the *i*th and (*i* + 4)th priority video data.

### 4.3 Comparison of UEP techniques in usage of wireless resources

An important aspect of wireless video streaming is how wireless resources with different protection levels (PLs) are utilized for streaming video data with different priority levels. In this section, the two previous and the proposed UEP techniques are compared in terms of how PHY layer resources are used. The number of PHY packets of a PL that are used for streaming the *p*th picture in the *g*th GOP is denoted as *N*(PL, *p*, *g*), where *p* and *g* denote the picture index and GOP index, respectively. Thus, the average number of PHY packets used for streaming a picture of *p*th priority can be calculated as follows:

$$\begin{array}{c} \bar{N}\left(\text{PL},p\right)=\frac{1}{G}\sum_{g=1}^{G}N\left(\text{PL},p,g\right) \end{array}$$ (8)

where G denotes the number of GOPs. We reiterate that the picture index *p* can also be considered as the source significance information (SSI), i.e., a smaller *p* indicates a higher significance of the picture. Tables 3–5 show the distribution of the PHY packets according to PL (the antenna reliability, MSBs or LSBs), and the source significance (picture index *p*) when the City video is considered. We note that the average numbers were rounded to the nearest integers in Tables 3–5. As smaller *p* indicates higher-priority video data and smaller PL denotes a resource with stronger protection, intuitive mapping is diagonal mapping from the upper left corner to the bottom right corner.

**Table 3:** $\bar{N}\left(\text{PL},p\right)$ in Previous multi-antenna UEP. Usage of PHY resources with different PLs for streaming of video data with different SSIs. Smaller PL and smaller *p* indicate stronger error protection and higher significance in terms of video quality, respectively.

PL\ *p*	1	2	3	4	5	6	7	8	9	10	11	12	13	14	15	16
1	134	3														
2	5	23	27	27	27	24	4									
3						4	22	28	28	27	24	4				
4											3	23	29	28	27	27
5	134	3														
6	5	23	27	27	27	24	4									
7						4	22	28	28	27	24	4				
8											3	23	29	28	27	27

**Table 4:** $\bar{N}\left(\text{PL},p\right)$ in Previous HQAM UEP. Usage of PHY resources with different PLs for streaming of video data with different SSIs.

PL\ *p*	1	2	3	4	5	6	7	8	9	10	11	12	13	14	15	16
1	69	13	14	14	13	12	2									
2	69	13	14	14	13	12	2									
3	69	13	14	14	13	12	2									
4	69	13	14	14	13	12	2									
5						2	11	14	14	14	14	14	14	14	14	13
6						2	11	14	14	14	14	14	14	14	14	13
7						2	11	14	14	14	14	14	14	14	14	13
8						2	11	14	14	14	14	14	14	14	14	13

**Table 5:** $\bar{N}\left(\text{PL},p\right)$ in Proposed HQAM-multi-antenna UEP. Usage of PHY resources with different PLs for streaming of video data with different SSIs.

PL\ *p*	1	2	3	4	5	6	7	8	9	10	11	12	13	14	15	16
1	137															
2	130	7														
3	10	46	53	27	2											
4			1	28	52	48	9									
5						8	44	55	30	1						
6								1	27	53	48	8				
7											7	46	58	27		
8														30	54	53

In the multi-antenna UEP technique, the I-picture uses 134 PHY packets of PL = 1 (the first reliable antenna MSBs), 5 PHY packets of PL = 2 (the second reliable antenna MSBs), 134 PHY packets of PL = 5 (the first reliable antenna LSBs), and 5 PHY packets of PL = 6 (the second most reliable antenna LSBs) as shown in Table 3. In the multi-antenna UEP technique, the video data of *p* = 1 are transmitted mostly using the wireless resources of the first and the fifth PLs. In HQAM UEP technique, the I-picture uses 69 PHY packets of the 1st through to the 4th PLs, as shown in Table 4. The remaining (137 − 69 = 68) resources of PL = 1 are used for streaming video data with *p* = 2, 3, ⋯, 7 as in the first row of Table 4.

From Table 5, it can be observed that the I-picture uses all 137 PHY packets of PL = 1, 130 PHY packets of PL = 2, and 10 PHY packets of PL = 3 in the proposed HQAM-multi-antenna UEP technique. We note that the numbers in Tables 3–5 are the average ones over GOPs; thus, $\bar{N}\left(2,1\right)=130$ does not indicate that 7 (= 137 − 130) PHY packets of PL = 2 are used for streaming less significant video data. Some I-pictures in the City video are not sufficiently large to require all PHY resources of PL = 1 and PL = 2. It can be observed that the City video I-picture does not use any PHY packets of PL = 5 (LSBs of the first reliable antenna) in the proposed technique. When compared with the proposed HQAM-multi-antenna UEP in which I-picture uses all PHY packets, which is protected the strongest among the PHY packets of 8 PLs, the HQAM UEP technique uses only 69 PHY packets of PL = 1 for the I-pictures, resulting in degraded video quality. Although only the I-picture is discussed in this section, the same discussion can be extended to the other pictures. It can be observed that the resource mapping of the proposed UEP is desirably more diagonal than that of the other two previous UEPs.

## 5 Simulation

In this section, the efficacy of the proposed HQAM-multi-antenna UEP technique is demonstrated with the aid of computer simulations. The 16-QAM constellation in Fig 2 was adopted with a spectral bandwidth of 20 MHz, a pilot tone pattern for OFDM systems with four antennas was used according to the standard [10], and linear interpolation was used for the channel estimation at the data subcarriers. The wireless channel is the ITU-R Pedestrian A model. A sub-band logical resource unit (SLRU) occupies 18 consecutive subcarriers and 6 OFDM symbol periods, including 16 pilots; hence, 18 × 6 − 16 = 92 subcarriers per SLRU are used for the data transmission. As 8 SLRUs are used for a physical frame with a period of 5 ms, the overall effective data transmission rate from four transmitter antennas is calculated as follows:

$$\begin{array}{c} \frac{92\times 8\times 4\text{bits}/\text{symbol}}{5\text{ms}}\times \frac{640-31}{1472}\times 4=998\text{kbps} \end{array}$$ (9)

where 640/1472 is the CTC channel coding rate (I_size_offset parameter in the standard is 20), and 31 bits of the CRC are considered.

The test video streams are 256 frames of City and Soccer videos with 4CIF resolution. The GOP size is 16 (1 I-picture and 15 P-pictures as in Figs 1(a) and 3(a)), the frame rate is 10 fps, the quantization parameters (QPs) of I and P pictures are the same as 28, and no B-pictures were used. The motion vector search range is ±16 pixels with a resolution of 1/4 pixel, the number of reference pictures is 1, and CABAC was used for entropy coding. The required data transmission rates are approximately 854.6 kbps and 852.2 kbps for City and Soccer videos, respectively. Consequently, the data transmission rate in Eq (9) is sufficient for these test videos.

### 5.1 PHY packet error performance

**Fig 4:**
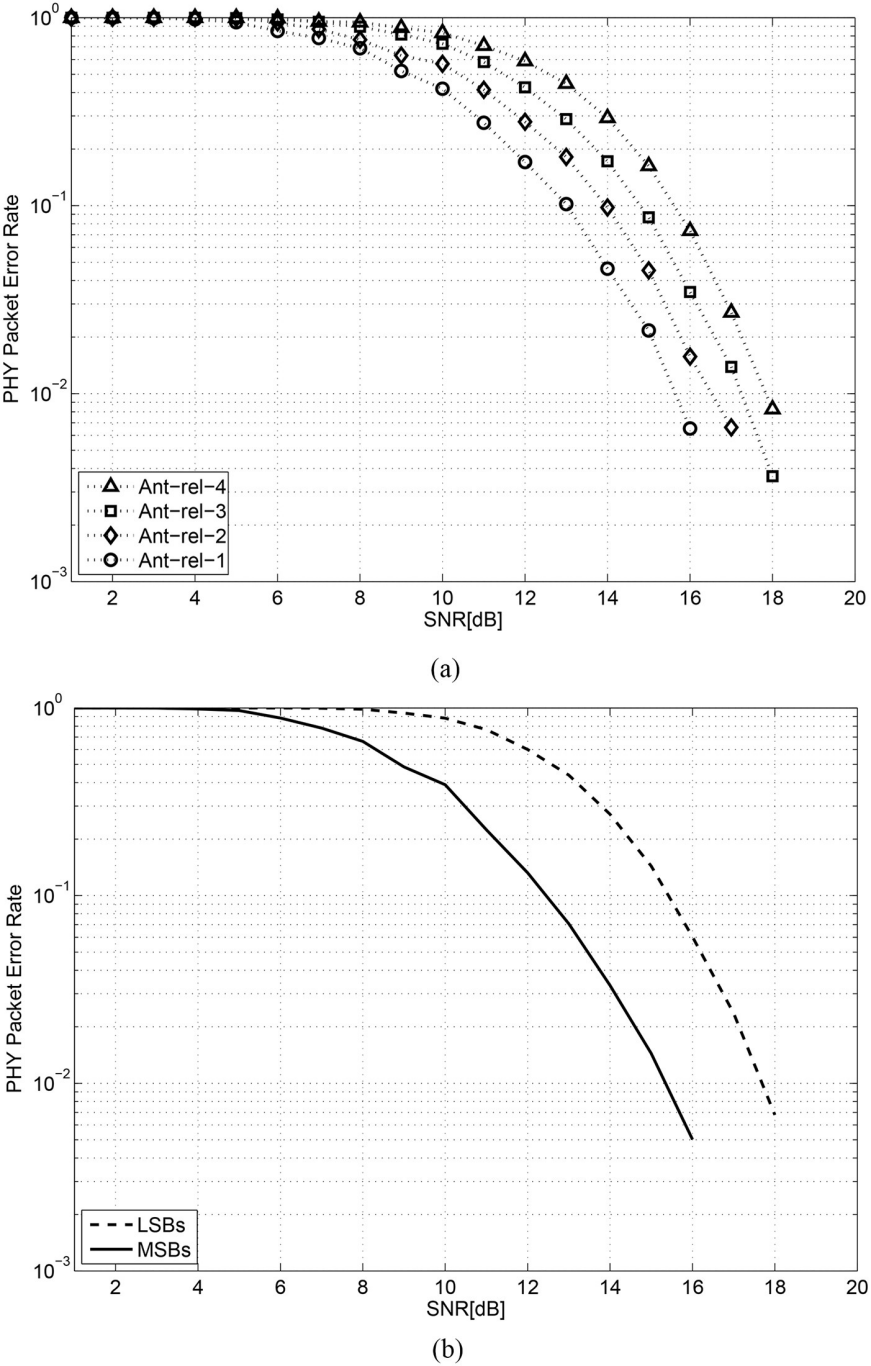
WirelessMAN-Advanced system PHY layer packet error rate achieved using previous works (a) Multi-antenna UEP; (b) HQAM UEP.

**Fig 5:**
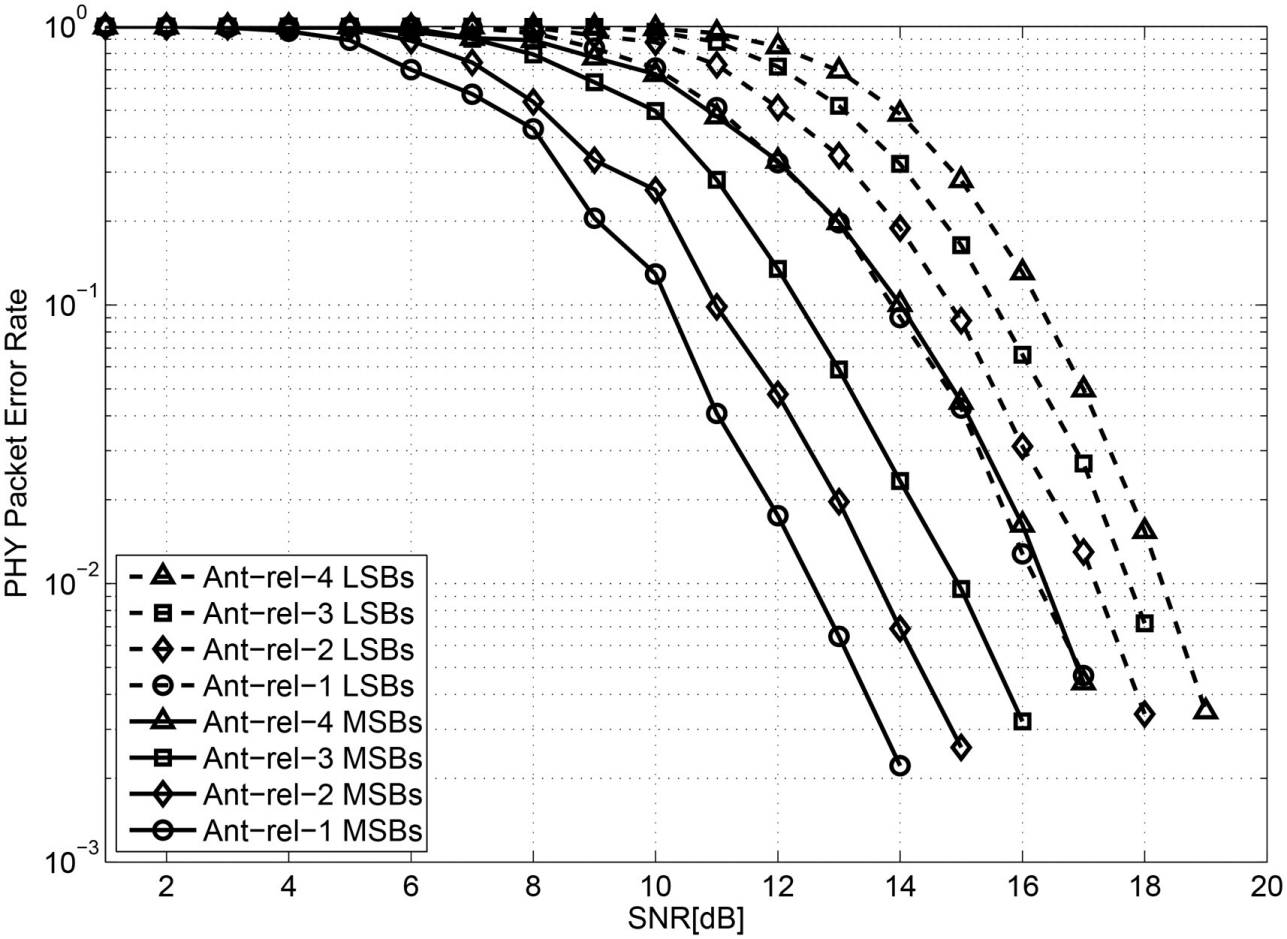
WirelessMAN-Advanced system PHY layer packet error rate achieved using the proposed HQAM-multi-antenna UEP.

Figs 4 and 5 show the physical layer packet error rates of IEEE802.16m systems for the two previous UEP techniques and the proposed UEP technique, respectively. In the multi-antenna UEP technique, UEP is achieved by the four different PLs of antennas as shown in Fig 4(a). In the HQAM UEP technique, two different PLs are implemented by the MSBs and LSBs of symbols regardless of antenna reliability as shown in Fig 4(b). The eight different PLs in the proposed UEP technique are shown in Fig 5. It can be observed that the performances of PL = 4 (the MSBs of the fourth reliable antenna) and PL = 5 (the LSBs of the first reliable antenna) are almost identical, which does not violate the assumed PLs in Table 2. Note that the average PHY packet error rates of the three UEP techniques are almost the same when they are averaged over all antennas and LSBs/MSBs, because the same joint signal detection technique and the same channel decoder are used for the three schemes. We note that a better UEP video streaming technique achieves higher video quality with a fixed overall PHY layer packet error rate, especially when the channel SNR is low.

When the target PHY error performance is fixed at 0.01, the most (least) reliable antenna in the multi-antenna UEP requires SNR of approximately 15.8 dB (17.9 dB). At the same target PHY error rate, an MSB (LSB) type PHY packet in the HQAM UEP requires SNR of approximately 15.4 dB (17.7 dB). In the proposed UEP technique, the most reliable combination (MSBs of the first reliable antenna) requires SNR of approximately 12.5 dB, which is the lowest among the three techniques. The least reliable combination (LSBs of the fourth reliable antenna) requires SNR of 18.2 dB, which is slightly higher than that of the LSB type packet in HQAM UEP and that of the most unreliable antenna in the multi-antenna UEP. It can be concluded that the proposed UEP technique protects video data of the highest priority stronger than the previous two UEP techniques. The purpose of this section is to show that a larger number of differentiated PLs enable more flexible and powerful UEP.

### 5.2 NAL unit NACK ratio

**Fig 6:**
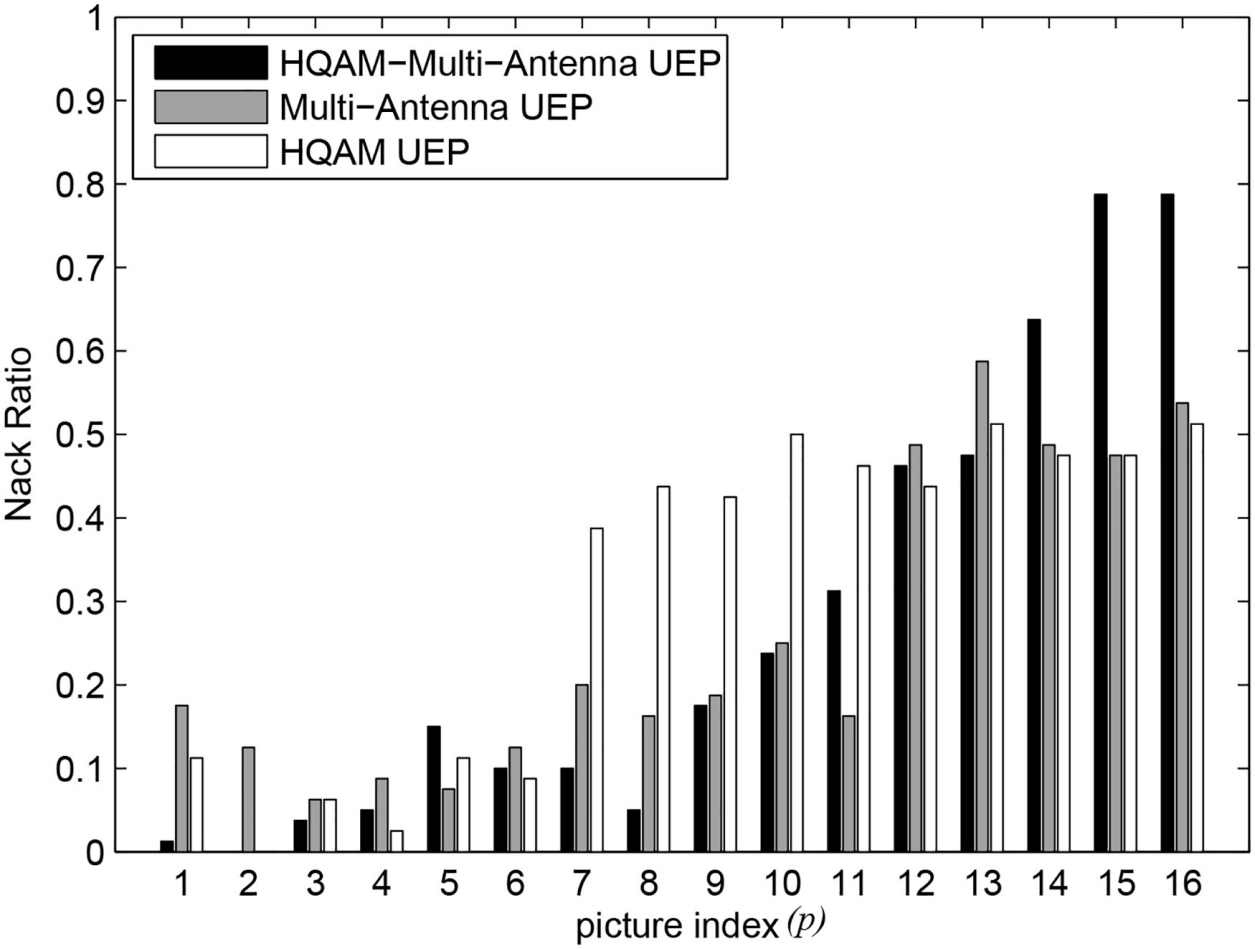
NACK ratios of the three UEP techniques for the City video when channel SNR is 18 dB.

Fig 6 compares the three UEP techniques in terms of average NACK ratios versus picture index. The City video was transmitted and reconstructed five times to facilitate calculation of the statistics. All three UEP techniques achieve lower NACK ratios for video data with higher priority, i.e., smaller *p* than for lower-priority video data. It can be observed that the NACK ratio of I-picture (*p* = 1) achieved using the proposed HQAM-multi-antenna UEP is the lowest among the three techniques. It can also be observed that the NACK ratio of the last picture (*p* = 16) achieved using the HQAM-multi-antenna UEP is the highest among the three techniques. Thus, it can be concluded that the proposed HQAM-multi-antenna UEP most severely sacrifices the video data of lower priority in order to protect video data with higher priority among the three techniques. The average NACK ratios (averaged over 16 pictures) are 0.2617, 0.3141, and 0.2734 for the multi-antenna UEP, HQAM UEP, and the proposed HQAM-multi-antenna UEP techniques, respectively, for the City video. The average NACK ratios of the three UEP techniques are more or less the same, but there is PSNR video quality improvement of 2 dB or 4 dB owing to the proposed technique at fixed channel SNRs.

### 5.3 Video quality performance

**Fig 7:**
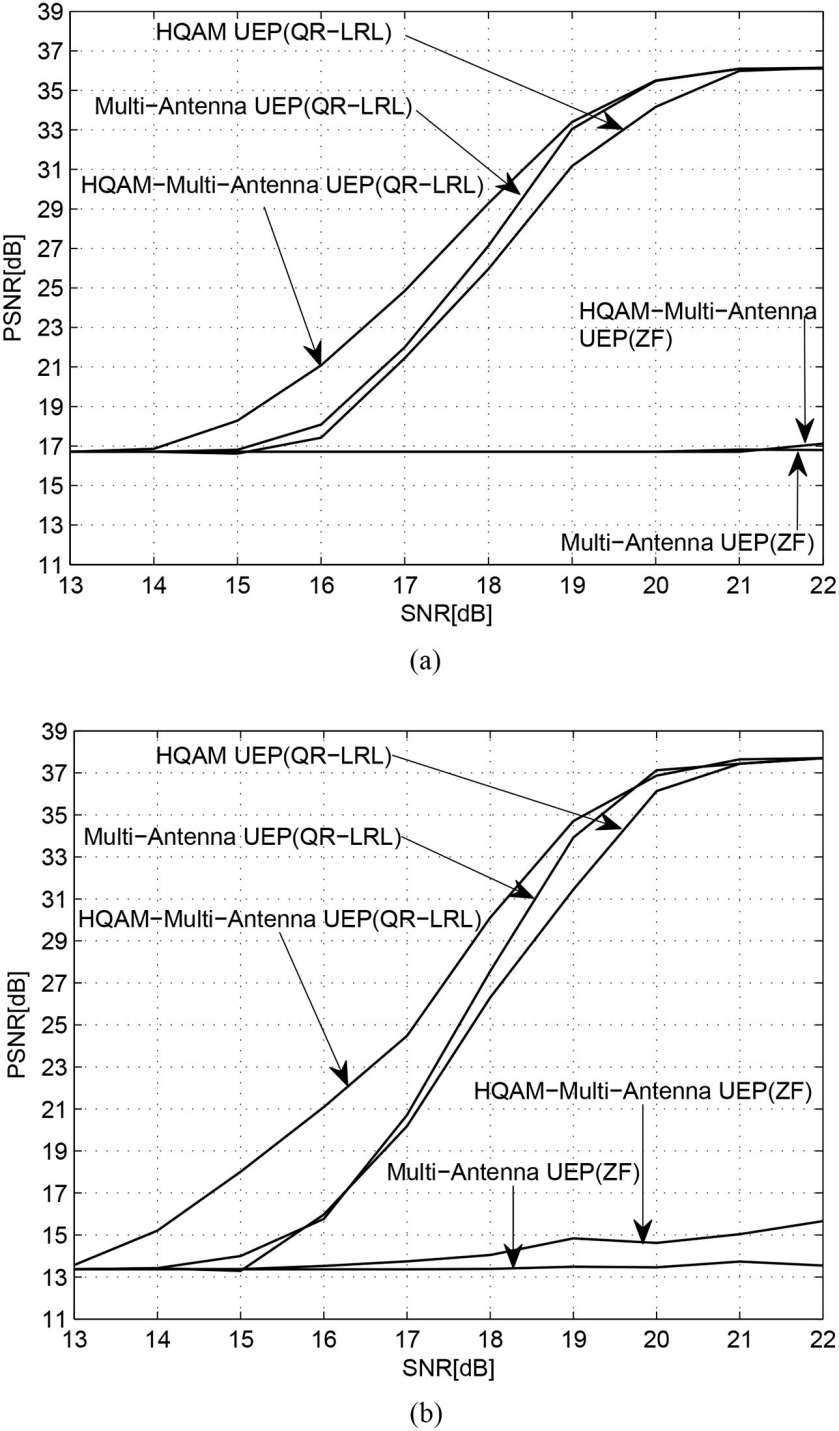
YPSNR performance versus channel SNRs for the previous multi-antenna UEP, previous HQAM UEP, and the proposed HQAM-multi-antenna UEP techniques. Also shown are the performances of linear-detection-based UEP techniques. (a) City video; (b) Soccer video.

Fig 7 compares the three UEP techniques in terms of the reconstructed video quality for various channel SNR values. As shown in Fig 7, the proposed UEP technique, which combines the HQAM UEP and the multi-antenna UEP, offers the highest PSNR performance among the three techniques for all channel SNR values and for both test videos. The PSNR improvement achieved using the proposed UEP technique is more significant for Soccer video especially when the channel SNR is low. Given that the numbers of differentiated PLs of HQAM UEP, multi-antenna UEP, and the proposed UEP are 2, 4, and 8, respectively, we can conclude that the video quality improves as the number of PLs increases. Fig 7 also shows the PSNR performance when a linear detection (ZF) method is adopted, because the HQAM-multi-antenna UEP technique can be combined with ZF detection. When ZF detection is assumed, post-detection SNR is used for the assessment of antenna reliability. The performance improvement with ZF, however, is observed to be limited mainly owing to the poor error performance of the linear detection techniques. From Fig 7, it can also be stated that the most significant factor in terms of video quality is the choice between linear detection and joint detection rather than the use of HQAM UEP and/or multi-antenna UEP.

## 6 Conclusion

In this paper, a novel UEP technique that uses both the previous multi-antenna UEP and the concept of HQAM UEP is proposed. As normal square QAM constellation, which is equivalent to HQAM with *α* = 1, is used in the proposed technique, the calculation of parameter *α* to achieve the desired error performance is not necessary; furthermore, QAM mapping at the transmitter side and the soft decision calculation at the receiver side need not be changed. Although standard square QAMs are used in the proposed technique, the term *HQAM* in the name of the proposed technique indicates that the UEP advantage of HQAM is retained. With a set of realistic simulations of the application-physical cross-layer in strict accordance with the IEEE802.16m standard and H.264/AVC video compression standard, it was shown that the proposed *HQAM-multi-antenna UEP* offers a significantly improved PSNR performance when compared with that of the previous UEP techniques. We also observed how wireless resources with different PLs are used for streaming videos with different significance levels in order to explain the improved video quality achieved using the proposed UEP. In an effort to even better improve the video quality than the proposed technique, a possible future work is to transmit pictures selectively and/or to allocate more power to a subset of selected transmit antennas.
